# Digital Phenotyping for Stress, Anxiety, and Mild Depression: Systematic Literature Review

**DOI:** 10.2196/40689

**Published:** 2024-05-23

**Authors:** Adrien Choi, Aysel Ooi, Danielle Lottridge

**Affiliations:** 1 School of Computer Science Faculty of Science University of Auckland Auckland New Zealand

**Keywords:** digital phenotyping, passive sensing, stress, anxiety, depression, PRISMA, Preferred Reporting Items for Systematic Reviews and Meta-Analyses, mobile phone

## Abstract

**Background:**

Unaddressed early-stage mental health issues, including stress, anxiety, and mild depression, can become a burden for individuals in the long term. Digital phenotyping involves capturing continuous behavioral data via digital smartphone devices to monitor human behavior and can potentially identify milder symptoms before they become serious.

**Objective:**

This systematic literature review aimed to answer the following questions: (1) what is the evidence of the effectiveness of digital phenotyping using smartphones in identifying behavioral patterns related to stress, anxiety, and mild depression? and (2) in particular, which smartphone sensors are found to be effective, and what are the associated challenges?

**Methods:**

We used the PRISMA (Preferred Reporting Items for Systematic Reviews and Meta-Analyses) process to identify 36 papers (reporting on 40 studies) to assess the key smartphone sensors related to stress, anxiety, and mild depression. We excluded studies conducted with nonadult participants (eg, teenagers and children) and clinical populations, as well as personality measurement and phobia studies. As we focused on the effectiveness of digital phenotyping using smartphones, results related to wearable devices were excluded.

**Results:**

We categorized the studies into 3 major groups based on the recruited participants: studies with students enrolled in universities, studies with adults who were unaffiliated to any particular organization, and studies with employees employed in an organization. The study length varied from 10 days to 3 years. A range of passive sensors were used in the studies, including GPS, Bluetooth, accelerometer, microphone, illuminance, gyroscope, and Wi-Fi. These were used to assess locations visited; mobility; speech patterns; phone use, such as screen checking; time spent in bed; physical activity; sleep; and aspects of social interactions, such as the number of interactions and response time. Of the 40 included studies, 31 (78%) used machine learning models for prediction; most others (n=8, 20%) used descriptive statistics. Students and adults who experienced stress, anxiety, or depression visited fewer locations, were more sedentary, had irregular sleep, and accrued increased phone use. In contrast to students and adults, less mobility was seen as positive for employees because less mobility in workplaces was associated with higher performance. Overall, travel, physical activity, sleep, social interaction, and phone use were related to stress, anxiety, and mild depression.

**Conclusions:**

This study focused on understanding whether smartphone sensors can be effectively used to detect behavioral patterns associated with stress, anxiety, and mild depression in nonclinical participants. The reviewed studies provided evidence that smartphone sensors are effective in identifying behavioral patterns associated with stress, anxiety, and mild depression.

## Introduction

### Background

Digital phenotyping is “the moment-by-moment quantification of the individual level human phenotype in situ using data from personal digital devices” [1]. Digital phenotyping applies the concept of phenotypes, in other words, the observable characteristics resulting from the genotype and environment, to conceptualize observable patterns in individuals’ digital data. In the last decade, digital phenotyping studies have been able to compare typical and atypical patterns in daily activities to correlate atypical behavior with negative emotions [2,3]. Behavioral patterns include variations in mobility, frequency of being in various locations, and sleep patterns. In smartphones, user data can be stored, managed, interpreted, and captured in enormous amounts [1,4,5]. This can be done actively or passively. Active data collection requires the user to self-report and complete surveys, whereas passive sensing collects data automatically without user input [5]. Most studies combine active and passive sensing to more accurately detect and predict behavioral abnormalities. Modern smartphone analytics can be used for the discovery of commonalities and abnormalities in user behavior. The ease of using passive sensing makes it an ideal data gathering method for mental health studies [6-8] and an ideal technique for assessing mental health [9].

Digital phenotyping has been successful in the early detection and prediction of behaviors related to neuropharmacology [10]; cardiovascular diseases [11]; diabetes [12]; and major severe injuries, such as spinal cord injury [13], motivating further adoption. Digital phenotyping has also proven useful for the detection of severe mental health issues, such as schizophrenia [14,15], bipolar disorder [16], and suicidal thoughts [17]. Digital phenotyping has been so successful for specialized, clinical populations that it is increasingly considered for mass market use with nonclinical populations. Digital phenotyping applications and software tools have been used to capture employee information, such as their screen time and clicking patterns [18]. However, there are not many digital phenotyping studies that have specifically examined the detection or prediction of stress, anxiety, and mild depression.

Individuals with stress, anxiety, and mild depression can develop chronic mental health symptoms that impact their mobility, satisfaction with life, and social interaction [19,20]. When these symptoms are not detected early, they worsen, and the impact is more significant [21-23], increasing the need for medication and hospitalization. This makes mild mental health symptoms a valid target for digital phenotyping, as its goal is to enable early detection and, subsequently, early treatment. Smartphones are increasingly ubiquitous [24], which makes them an optimal platform for digital phenotyping. We constrained our systematic literature search to the more challenging problem of the detection of mild mental health symptoms using only smartphone sensors and excluded studies that used additional wearable sensors. In general, we believe that additional wearables might increase the effectiveness of digital phenotyping in detecting stress, anxiety, and mild depression. Given the ubiquity of smartphones, we aimed to answer the following question: what is the effectiveness of digital phenotyping using smartphone sensors in detecting stress, anxiety, and mild depression?

### Objectives

The objective of this systematic literature review was to better understand the current uses of digital phenotyping and results of using digital phenotyping for the detection and prediction of mild behavioral patterns related to stress, anxiety, and mild depression. The 2 research questions this review sought to answer were as follows:

1. What is the evidence of the effectiveness of digital phenotyping using smartphones in identifying behavioral patterns related to stress, anxiety, and mild depression?
2. In particular, which smartphone sensors are found to be effective, and what are the associated challenges?

For these research questions, we considered statistically significant associations between sensor patterns and behavioral patterns as evidence of effectiveness.

## Methods

### Type of Studies

**Figure 1:**
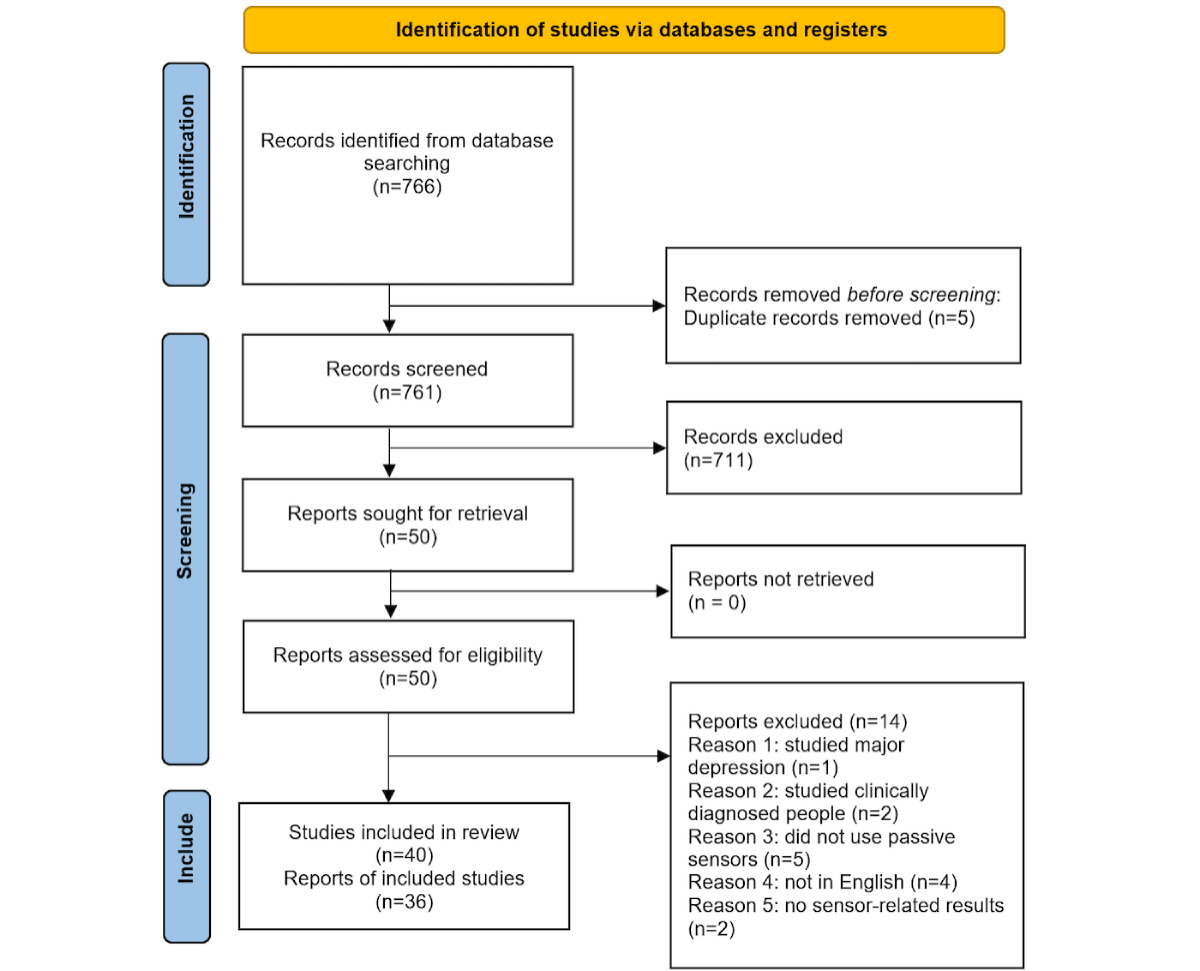
Systematic literature reviewing process and search results with the PRISMA (Preferred Reporting Items for Systematic Reviews and Meta-Analyses) diagram.

This review followed the PRISMA (Preferred Reporting Items for Systematic Reviews and Meta-Analyses) guidelines [25] (Multimedia Appendix 1). Figure 1 shows the reviewing process and search results. In the first round of screening studies, 1 author excluded studies that were not relevant to the research questions. Another author reran the queries for confirmation. Studies were included in this review if they were conducted to measure and detect stress, anxiety, or mild depression, even if they included other variables, such as job performance, promotion, or discrimination. We included studies in which data were collected through smartphones with an iOS (Apple Inc) or Android (Google LLC) operating system. Data collected through wearable devices were excluded. We included studies in which the participants were adults aged ≥18 years and were from a nonclinical population. Studies conducted with nonadult participants (eg, teenagers and children) were excluded. Given our research questions, if the studies’ participants had or had had any severe mental health disorder, such as schizophrenia, bipolar disorder, or psychosis, they were not included. We also excluded personality and character measurement and phobia studies. The primary research language was English. The studies included were conducted from September 2010 to September 2023. Peer-reviewed conference articles and journal articles were included. The data we wished to extract were the study aim, data collected, operating system in the smartphone used for data collection, behavioral patterns identified, surveys used for verification, and sample size. A total of 3 authors reviewed the studies independently to extract data and confirm the extracted data. After the first round of data extraction, 1 author re-examined the studies to extract the predictive modeling used. These data are presented in the *Results* section. We noticed that participants in the included studies fell into 1 of 3 major groups (ie, students, adults, and employees). We refer to the participants of the studies that recruited adults enrolled in universities as “students,” participants of the studies that recruited adults unaffiliated to any particular organization as “adults,” and participants of the studies that recruited adults employed at a particular organization as “employees.”

### Search Strategy

A total of 3 databases were queried: Web of Science, ACM, and PubMed. PubMed is a medicine-based database, ACM is a technology-based database, and Web of Science is a cross-domain database. The search query was the same for the 3 platforms: “digital phenotyping” OR “passive sensing” AND (stress OR anxiety OR ((mild OR moderate) AND depression)).

## Results

### Duration

The study length varied from 10 days [26] to 3 years [27]. One study [28] conducted in-depth interviews with students lasting an average of 4.5 hours per person, and another study was a controlled laboratory study [29]. These 2 studies are not presented in Table 1. In the studies conducted with students, a semester or spring or winter term was a common duration. The studies with general nonclinical adult populations were typically longer than those with students.

**Table 1:** Duration of the reviewed studies (N=38; 2 studies are excluded, as 1 [28] is interview based and the other [29] is a controlled laboratory study).

Study, year	Length of the study (d)
Adams et al [26], 2014	10
Cai et al [30], 2018	14
Boukhechba et al [31], 2018	14
Di Matteo et al [32], 2021	14
Jacobson et al [33], 2020	16
Wen et al [34], 2021	21
Melcher et al [35], 2023	28
Fukuzawa et al [36], 2019	28
Rashid et al [37], 2020	35
Zakaria et al [38], 2019	35
DaSilva et al [39], 2019	43
Nepal et al [40], 2020	60
Saha et al [41], 2019	68
Morshed et al [42], 2019	70
Acikmese et al [43], 2019	70
Zakaria et al [38], 2019	81
Zakaria et al [38], 2019	81
Boukhechba et al [44], 2017	98
Tseng et al [45], 2016	98
Morshed et al [42], 2019	98
Xu et al [46], 2019	106
Chikersal et al [47], 2021	112
Meyerhoff et al [48], 2021	112
Xu et al [46], 2019	113
Rhim et al [49], 2020	121
Wang et al [50], 2018	121
Currey and Torous [51], 2022	147
Di Matteo et al [52], 2021	153
Sefidgar et al [53], 2019	153
Mendu et al [54], 2020	153
Pratap et al [55], 2017	181
Mirjafari et al [56], 2019	260
Currey et al [57], 2023	336
Huckins et al [58], 2020	458
Mack et al [59], 2021	458
Xu et al [60], 2023	458
Nepal et al [61], 2022	730
Servia-Rodríguez et al [27], 2017	1095

### Number of Participants

The number of participants ranged from a minimum of 7 adults [26] to a maximum of 18,000 adults [27]. Apart from the 3-year longitudinal study with 18,000 participants [27], the average number of participants was 129.4 (SD 184.01). We observed a pattern of attrition, where the number of participants who completed the study was lower than the number of the participants recruited. The number of participants reported in this review is the final sample size. For example, one of the studies [52] recruited 112 participants, of whom 84 (75%) completed the study. In the study by Pratap et al [55], there was a drastic drop in participants, with only 359 (30.42%) of the 1180 enrolled participants completing the study. Another significant drop was seen in the study by Nepal et al [40], where 750 participants were interested in the research, whereas only 141 (18.8%) of them completed the study. Some studies were less affected; for example, 86 participants started the study by Rhim et al [49], and 78 (91%) completed it.

### Publication Years of the Studies

Although the query started with the year 2010, the earliest publication was from 2014 [26], extending to articles published as of April 2023 [35]. Over the years, the interest in detecting and predicting stress, anxiety, and mild depression in the nonclinical population has increased (Table 2).

**Table 2:** Number of reviewed reports (N=36) by year.

Year	Publication, n (%)
2014	1 (3)
2016	1 (3)
2017	3 (8)
2018	4 (11)
2019	10 (28)
2020	6 (17)
2021	6 (17)
2022	2 (6)
2023	3 (8)

### Studies With the iOS and Android Operating Systems

The Android operating system was more common than iOS. Among the 40 included studies, only 2 (5%) were compatible with only iOS [29,51]. A total of 27 (68%) studies were available for both iOS and Android [26,28,30,34,35,37-42,45-47,50,53-61]. A total of 11 (28%) studies were for only Android users [27,31-33,36,42-44,48,49,52]. The reasons identified for the use of the Android operating system were that it has more freedom to capture more modalities, such as keyboard typing and use of apps, and that Android devices enable apps to run more easily in the background [49].

### Studies With Students

Table 3 presents the data extracted from the studies that were conducted with student populations. The average length of the studies with students was 158.6 (SD 176.4) days. The average number of participants was 137.3 (SD 152.1). There were significantly more studies with students than studies with employees or general adults. The sample sizes of the studies with students were similar to those of the studies with adults but smaller than those of the studies with employees. In the studies with students, various passive sensors were used, and some were found to be effective for detection, prediction, or both.

Of the 28 studies with students, 23 (82%) used machine learning models for prediction. A total of 12 studies (43%) [30,31,33,37,38,44,46,47,54] used decision tree–based methods, and 9 studies (32%) [37,39,42,49-51,57,58] used regression-based methods. A total of 3 (11%) studies conducted in recent years [43,60,61] used deep neural networks because of their enhanced ability to discern underlying patterns in large unstructured data sets. Tree-based models have the best performance when trained with structured data, and the reported studies mostly used tree-based models and structured data. Among the 28 studies, 2 studies [57,60] conducted in 2023 addressed the generalizability of their proposed detection method and verified its applicability across students from various years, classes, and institutions. Two (7%) studies [42,43] in Table 3 used the StudentLife data set [62]. Each study contributed substantial original analyses including different behavioral patterns and was considered a “study” in this systematic review. Entries with “N/A” in the predictive modeling column indicate that the study did not involve any attempts to predict future occurrences. However, these studies may still contain statistical analyses as part of their research approach. Overall, students who experienced depression, anxiety, and stress visited fewer locations [39,44,50,58-60] and were more sedentary [47,50,58-60]. Depression was also associated with shorter or irregular sleep [35,46,47,50,52,59,60] and accrued phone use [46,47,50,51,58-60].

**Table 3:** Summary of the reviewed studies with student participants.

Study, year	Aim	Data collected	Operating system	Behavioral patterns	Predictive modeling	Verification surveys	Sample size, n
Huckins et al [58], 2020	Understand how students’ behavioral health and mental health are affected by the COVID-19 pandemic	GPS, accelerometer, phone lock and unlock, and light sensor data	iOS (Apple Inc) and Android (Google LLC)	At the start of the COVID-19 pandemic, students were more depressed and anxious, used their phones more, visited fewer locations, and spent more time sedentary. Depression and stress were associated with increasing COVID-19–related news coverage.	Linear regressors were used to inspect how behavioral changes were affected by COVID-19 news reports.	PHQ^a^-4	217 students
Melcher et al [35], 2023	Understand how behavioral patterns correlate with mental health for students during the COVID-19 pandemic	GPS, accelerometer, call log, and phone use data	iOS and Android	Individuals with more irregular sleep patterns had worse sleep quality and were experiencing more depression and more stress than those with consistent sleep patterns.	N/A^b^	PHQ-9, DASS^c^, SIAS^d^, GAD-7^e^, PQ^f^, PSS^g^, PSQI^h^, BASIS^i^, SF^j^-36, SFS^k^, Flourishing Scale, CGI^l^, HDRS^m^, CAS^n^, HAI^o^, and UCLA^p^-Loneliness Scale	100 students
Jacobson et al [33], 2020	Predict social anxiety symptom severity and discriminate between depression, negative affect, and positive affect	Accelerometer, call log, and SMS text message data	Android	Measures of SMS text message and call response time discriminated among depression, negative affect, and positive affect. Accelerometer patterns suggested that persons with low social anxiety walked at a steady pace, whereas persons with high social anxiety walked more quickly with more irregularity.	XGBoost^q^ with LOOCV^r^ was used to predict social anxiety symptom severity.	SIAS, DASS-21, and PANAS^s^	59 students
DaSilva et al [39], 2019	Predict stress	GPS, accelerometer, phone lock and unlock, microphone, and light sensor data	iOS and Android	Students with stress were more likely to spend less time in campus food locations and more time in schoolwork locations. Students with stress traveled less, engaged in fewer conversations, and were in quieter environments during evenings.	Penalized generalized estimating equations were used to prune features and fit a marginal regression model to predict stress.	MPSM^t^	94 students
Acikmese and Alptekin [43], 2019	Predict stress level	Accelerometer, microphone, Bluetooth, light sensor, phone lock and unlock, phone charge, and app use data (GPS and Wi-Fi data were collected but not used)	Android	Students were successfully categorized as stressed or nonstressed using the measured sensors.	LSTM^u^, CNN^v^, and CNN-LSTM were used to classify stress, with LSTM yielding the best accuracy.	Self-reported stress	48 students
Rooksby et al [28], 2019	Understand students’ perspectives about digital phenotyping	GPS, phone lock and unlock, phone charge, battery, microphone, Bluetooth, light sensor, SMS text message, email, app use, call log, camera, and keyboard data	iOS and Android	None of the results related sensors to symptoms of depression or anxiety. Students have privacy concerns regarding the use of app use logs, Bluetooth data, call logs, camera data, keyboard data, and microphone data but not regarding the use of battery, or light sensor. Students had privacy concerns with the use of SMS text message content but not with counts of messages.	N/A	PHQ-9, GAD-7, and WEMWBS^w^	15 students
Chikersal et al [47], 2021	Predict postsemester depressive symptoms	GPS, accelerometer, Bluetooth, Wi-Fi, phone use, call log, and microphone data	iOS and Android	Depression was predicted by participants’ social context in the afternoons and evenings, phone use throughout the day, long periods without exercise, periods of disturbed sleep at night, and time spent outdoors.	Trained an ensemble classifier with the outputs from models containing features from 1 sensor, with different setting combinations.	BDI^x^-II	138 students
Morshed et al [42], 2019	Predict mood instability	Accelerometer, microphone, Bluetooth, light sensor, Wi-Fi, GPS, phone lock and unlock, and phone charge data	Android	Mood instability was negatively correlated with the duration of sleep, the number of conversations, the amount of activity, and outdoor mobility.	Ridge regression with regularization was used to infer mood instability score.	EMAs^y^, PAM^z^, and PANAS	48 students
Zakaria et al [38], 2019	Detect depression and stress	Wi-Fi data	iOS and Android	Students with severe stress spent significantly less time on campus and were less involved in work-related activities than students with normal stress. Students with severe stress were more involved in these activities at the start of the semester, but the involvement decreased over time.	The random forest stress model with domain-specific features achieved the best result, with feature sets changed every 6 days.	PSS-4, PHQ-8, and BFI^aa^	62 students
Zakaria et al [38], 2019	Detect depression and stress	Wi-Fi data	iOS and Android	Same patterns as those mentioned earlier.	The random forest model that excluded domain-specific features achieved the best result, with feature sets changed every 6 days.	PSS-4, PHQ-8, and BFI	11 students
Zakaria et al [38], 2019	Detect depression and stress	Wi-Fi data	iOS and Android	Same patterns as those mentioned earlier.	The best model is a random forest model with the neuroticism score added as an additional feature, with sensor data sets calculated with a 6-day interval.	PSS-4, PHQ-8, and BFI	35 students
Wang et al [50], 2018	Predict depression	Light sensor, GPS, accelerometer, microphone, screen on and off, and phone lock and unlock data	iOS and Android	Students who experienced depression had more irregular sleep patterns, used their phones more at study places, spent more time stationary, and visited fewer locations.	LASSO^ab^ regression was used to predict presurvey and postsurvey PHQ-9 scores.	PHQ-4 and PHQ-8	83 students
Exposito et al [29], 2018	Detect stress	Keyboard 3D touch data	iOS	Students’ typing pressure increased under stress.	N/A	Self-reported stress	11 students
Rhim et al [49], 2020	Detect subjective well-being and stress	Accelerometer, GPS, screen on and off, app use, and notification data	Android	Lower subjective well-being was associated with more time spent on campus, more time spent stationary, increased phone use in the evenings, and more expenses.	Hierarchical regression models were used to predict subjective well-being.	COMOSWB^ac^, PHQ, SAS^ad^, PPC^ae^, and BFI	78 students
Sefidgar et al [53], 2019	Detect stress, anxiety, and gender discrimination	Accelerometer, GPS, phone lock and unlock, screen on and off, and call log data	iOS and Android	Students who experienced discrimination became more physically active; their phone use increased in the morning, they had more calls in the evening, and they spent more time in bed on the day of the discrimination.	Linear regression was used to predict long-term changes in mental health states; hierarchical linear modeling was used for short-term prediction.	UCLA Loneliness Scale, SSS^af^, MAAS^ag^, ERQ^ah^, BRS^ai^, PSS, CES-D^aj^, STAI^ak^, and self-reported affect and fairness of treatment	176 students
Cai et al [30], 2018	Detect state affect, stress, anxiety, and depression	Accelerometer, GPS, call log, and SMS text message data	iOS and Android	Negative emotions were related to geographical locations, but this was affected by personal routines and preferences, for example, liking cinema theatres. On Fridays and Saturdays, students reported less negative states.	Compared support vector machine, random forest, and XGboost with LOSOCV^al^ and LOOCV to predict negative affect. The best model was support vector machine with LOOCV.	SIAS and self-reported affect (EMAs)	220 students
Boukhechba et al [31], 2018	Predict response rate and latency to EMA	GPS, call log, accelerometer, and SMS text message data	Android	None of the results related sensors to symptoms of depression or anxiety.	Used random forest, support vector machine, and a multilayer perceptron of 1 hidden layer with LOOCV to predict the compliance rate of EMA responses.	Self-reported affect (EMAs)	65 students
Xu et al [46], 2019	Detect depression	Accelerometer, battery or charge, Bluetooth, call log, screen, location, and phone lock and unlock data	iOS and Android	Students who experienced depression had more disturbed sleep patterns and more phone interactions than students who did not experience depression.	AdaBoost^am^ with decision tree–based components achieved the best performance when features were hybrid (contextually filtered + unimodal).	BDI-II	138 students
Xu et al [46], 2019	Detect depression	Accelerometer, battery or charge, Bluetooth, call log, screen, location, and phone lock and unlock data	iOS and Android	Same patterns as those mentioned earlier.	AdaBoost with decision tree–based components achieved a similar result to majority-based baseline predictors.	BDI-II	212 students
Boukhechba et al [44], 2017	Predict social anxiety	GPS, call log, and SMS text message data	Android	Students who experienced high social anxiety may be more likely to buy food so they can eat at home; they tended to visit fewer places and had a narrower range of activities.	Decision tree was used to predict SAS.	SIAS	54 students
Rashid et al [37], 2020	Predict social anxiety and evaluate the effectiveness of imputation methods in handling missing data	GPS, pedometer, accelerometer, call log, and SMS text message data	iOS and Android	The level of social anxiety was predicted, but there were no specific patterns relating sensors to symptoms of social anxiety.	Evaluated 7 predictive models: linear regression, decision tree, XBboost, lightGBM^an^, random forest, MERF^ao^, and CatBoost.	SIAS and self-reported dimensions of social anxiety	80 students
Mendu et al [54], 2020	Explore the relationships among private social media messages, personality traits, and symptoms of mental illness	Facebook (Meta Platforms, Inc) private messages	iOS and Android	Students who experienced anxiety received responses later, had more night-time communications, talked less about games and sports, and used more plural pronouns.	Used random forest classifier to select features and support vector machine with LOOCV to predict each psychological measure binarily.	STAI, UCLA Loneliness Scale, and TIPI^ap^	103 students
Tseng et al [45], 2016	Detect stress and its relationship with academic performance	Location, activity, step count (iOS only), audio, accelerometer (iOS only), device use, charging event, battery, light (Android only), SMS text message (Android only) and call (Android only) data and data about currently running apps (Android only)	iOS and Android	Students slept less during examination periods and more during breaks; they felt more stressed during the breaks and examination periods; sensor data were able to capture different routines during weekdays, weekends, and breaks.	N/A	PSQI, ESS^aq^, MCTQ^ar^, PROMIS^as^-10, BHM^at^-20, CD-RISC^au^, Flourishing Scale, Perceived Stress Scale, BFI, PHQ-8, and UCLA Loneliness Scale	22 students
Mack et al [59], 2021	Understand the association between behavioral and mental health and the COVID-19 pandemic	GPS, accelerometer, phone lock and unlock, and light sensor data	iOS and Android	During the COVID-19 pandemic, students experienced more depression and anxiety and increased sedentary time and phone use, whereas sleep and the number of locations visited decreased.	N/A	PHQ-4 and EMAs	217 students
Xu et al [60], 2023	Evaluate the cross–data set generalizability of depression detection	GPS, accelerometer, phone lock and unlock, Bluetooth, Wi-Fi, call log, microphone, gyroscope, and light sensor data	iOS and Android	Individuals who experienced depression had shorter sleep duration, had more interrupted sleep, had more frequent phone locks and unlocks, spent more time at home, were more sedentary, had fewer physical activities, visited fewer uncommon places, and had more consistent mobility patterns.	A multitask learning model with the 1D-CNN^av^–based embedding, fully connected layers for reordering and classification.	Weekly surveys on self-reported depression symptoms and affect, BDI-II, and PHQ-4	534 students
Nepal et al [61], 2022	Explore the association between students’ COVID-19 concerns and behavioral and mental health	GPS, accelerometer, phone lock and unlock, light sensor, and phone use data	iOS and Android	Heightened COVID-19 concerns correlated with increased depression, anxiety, and stress. No specific results relating sensors to symptoms of depression, anxiety, or stress were observed.	Evaluated different deep learning models in terms of their classification of COVID-19 concerns: CNN, InceptionTime, MCDCNN^aw^, ResNet^ax^, multilayer perceptron, TWIESN^ay^, LSTM, and FCNN^az^; FCNN performed the best, with an AUROC^ba^ score of 0.7.	Self-reported affect and PHQ-4	180 students
Currey and Torous [51], 2022	Predict survey results on mental health from passive sensors	GPS, accelerometer, call, and screen time data	iOS	Individuals at higher risks of psychosis spent less time at home. Individuals who were lonelier had longer sleep duration and fewer calls. Individuals who experienced stress or depression had longer outgoing calls.	Logistic regression was used to predict survey scores.	PHQ-9, GAD-7, PSS, UCLA Loneliness Scale, PQ-16, and PSQI	147 students
Currey et al [57], 2023	Explore the cross–data set generalizability of symptom improvement based on the surveys	GPS, accelerometer, and screen time data	iOS and Android	Logistic regression was able to predict changes in mood across 2 data sets of student participants. No results relating sensors to symptoms of depression or anxiety were observed.	Logistic regression was used to predict weekly score improvement from both active and passive features.	PHQ-9, GAD-7, PSS, UCLA Loneliness Scale, PSQI, PQ-16, and DWAI^bb^	698 students

^a^PHQ: Patient Health Questionnaire.

^b^N/A: not applicable.

^c^DASS: Depression Anxiety Stress Scales.

^d^SIAS: Social Interaction Anxiety Scale.

^e^GAD-7: Generalized Anxiety Disorder Scale-7.

^f^PQ: Prodromal Questionnaire.

^g^PSS: Perceived Stress Scale.

^h^PSQI: Pittsburgh Sleep Quality Index.

^i^BASIS: Behavior and Symptom Identification Scale.

^j^SF: Short Form Health Survey.

^k^SFS: Social Functioning Schedule Scale.

^l^CGI: Clinical Global Impressions Scale.

^m^HDRS: Hamilton Depression Rating Scale.

^n^CAS: Coronavirus Anxiety Scale.

^o^HAI: Health Anxiety Inventory.

^p^UCLA: University of California, Los Angeles.

^q^XGBoost: extreme gradient boosting.

^r^LOOCV: leave-one-out cross validation.

^s^PANAS: Positive and Negative Affect Schedule.

^t^MPSM: Mobile Photographic Stress Meter.

^u^LSTM: long short-term memory.

^v^CNN: convolutional neural network.

^w^WEMWBS: Warwick-Edinburgh Mental Well-Being Scale.

^x^BDI: Beck Depression Inventory.

^y^EMA: ecological momentary assessment.

^z^PAM: Patient Activation Measure.

^aa^BFI: Big Five Inventory.

^ab^LASSO: least absolute shrinkage and selection operator.

^ac^COMOSWB: Concise Measure of Subjective Well-Being.

^ad^SAS: Sport Anxiety Scale.

^ae^PPC: Perceived Personal Control.

^af^SSS: Social Support Scale.

^ag^MAAS: Mindful Attention Awareness Scale.

^ah^ERQ: Emotion Regulation Questionnaire.

^ai^BRS: Brief Resilience Scale.

^aj^CES-D: Center for Epidemiological Studies-Depression.

^ak^STAI: State Trait Anxiety Inventory.

^al^LOSOCV: leave-one-subject-out cross validation.

^am^AdaBoost: adaptive boosting.

^an^LightGBM: light gradient boosting machine.

^ao^MERF: mixed-effects random forest.

^ap^TIPI: Ten-Item Personality inventory.

^aq^ESS: Epworth Sleepiness Scale.

^ar^MCTQ: Munich Chronotype Questionnaire.

^as^PROMIS: Patient-Reported Outcomes Measurement Information System.

^at^BHM: Behavioral Health Measure.

^au^CD-RISC: Connor-Davidson Resilience Scale.

^av^1D-CNN: 1-dimensional convolutional neural network.

^aw^MCDCNN: multi-channel deep convolutional neural network.

^ax^ResNet: residual network.

^ay^TWIESN: time warping invariant echo state network.

^az^FCNN: fully convolutional neural network.

^ba^AUROC: area under the receiver operating characteristic curve.

^bb^DWAI: Digital Working Alliance Inventory.

### Studies With Adults

Table 4 presents the data extracted from the studies conducted with the general adult population. The average study duration was 201.6 (SD 367) days. Apart from a 3-year longitudinal study with 18,000 participants, the average number of participants was 123.4 (SD 139.8). Of the 8 studies with adults, 2 (25%) [32,52] were conducted with the same set of participants. A total of 3 (38%) studies used predictive modeling, with regression-based models being the most common [34,36,52], and 1 (12%) study identified gender differences in behavioral patterns [27]. Overall, the research with adults showed that GPS, accelerometer, ambient audio, and illuminance data related to individuals’ emotional state. Adults with depression were less likely to leave home and were less physically active, whereas adults who were socially anxious were more active and left their home more often but avoided going to places where they needed to socially interact.

**Table 4:** Summary of the reviewed studies with adult participants.

Study, year	Aim	Data collected	Operating system	Behavioral patterns	Predictive modeling	Verification surveys	Sample size, n
Di Matteo et al [32], 2021	Understand whether ambient speech correlates with social anxiety, generalized anxiety, and depressive symptoms	Microphone data	Android	Generalized anxiety and depression were correlated with reward-related words. Social anxiety was correlated with vision-related words.	N/A^a^	LSAS^b^, GAD-7^c^, PHQ^d^-8, and SDS^e^	86 Canadian adults
Di Matteo et al [52], 2021	Predict general anxiety disorder, social anxiety disorder, and depression	GPS, microphone, screen on and off, and light sensor data	Android	Depression and social anxiety were associated with increased screen use. Depression was associated with sleep disturbance and death-related word features.	A total of 3 logistic regression models were used to predict social anxiety disorder and generalized anxiety disorder with repeated k-fold cross validation.	LSAS, GAD-7, PHQ-8, and SDS	84 Canadian adults
Wen et al [34], 2021	Detect impulsive behavior, positivity, and stress	Call log, phone lock and unlock, and phone charging data	iOS and Android	Impulsivity was correlated with increased phone use and screen checking.	Used LASSO^f^ regularization to first select features and trained a linear regression model to estimate trait impulsivity scores.	BIS^g^-15, UPPS^h^, PAM^i^, and self-reported feelings	26 adults
Fukazawa et al [36], 2019	Predict anxiety levels and stress	Light sensor, gyroscope, accelerometer, and app use data	Android	Anxiety was higher from Monday to Thursday than on Friday and Saturday. Increased anxiety was associated with decreased mobility. During mild exercise, anxiety was reduced.	Used linear classifier by LASSO and XGBoost^j^ to classify the change of anxiety.	STAI^k^	20 adults
Pratap et al [55], 2017	Detect depression	GPS, call log, and SMS text message data	iOS and Android	None of the results related sensors to symptoms of depression.	N/A	PHQ-2 and PHQ-9	359 Hispanic or Latino adults
Adams et al [26], 2014	Detect stress level	Microphone data	iOS and Android	Stress can be recognized from pitch, speaking speed, and vocal energy.	N/A	PANAS^l^, PSS^m^-14, MAAS^n^, and self-reported affect	7 adults
Meyerhoff et al [48], 2021	Detect anxiety and depression	GPS, call log, app use, and SMS text message data	Android	Changes in the number of locations visited and social activity duration were associated with depression. Time spent at exercise locations was positively correlated with changes in depressive symptoms.	N/A	GAD-7, PHQ-8, and SPIN^o^	282 adults
Servia-Rodríguez et al [27], 2017	Predict mood	GPS, Wi-Fi, cell tower, accelerometer, microphone, SMS text message, and call data	Android	A strong correlation was identified between daily routines and users’ personality, well-being perception, and other psychological variables; the participants who were the most emotionally stable tended to be more active, stayed in more noisy places, and texted less than participants who were unstable.	Used stacked RBMs^p^ to classify moods.	Big-5 personality test, self-reported mood, and self-reports of locations	18,000 adults mainly

^a^N/A: not applicable.

^b^LSAS: Liebowitz Social Anxiety Scale.

^c^GAD-7: Generalized Anxiety Disorder Assessment-7.

^d^PHQ: Patient Health Questionnaire.

^e^SDS: Sheehan Disability Scale.

^f^LASSO: least absolute shrinkage and selection operator.

^g^BIS: Barratt Impulsiveness Scale.

^h^UPPS: Impulsive Behavior Scale.

^i^PAM: Patient Activation Measure.

^j^XGBoost: extreme gradient boosting.

^k^STAI: State Trait Anxiety Inventory.

^l^PANAS: Positive and Negative Affect Schedule.

^m^PSS: Perceived Stress Scale.

^n^MAAS: Mindful Attention Awareness Scale.

^o^SPIN: Social Phobia Inventory.

^p^RBM: Restricted Boltzmann Machine.

### Studies With Employees

Table 5 presents the data extracted from the studies that were conducted with employees. Among the 4 studies with employees, 1 (25%) study recruited its own participants [56], and the other 3 (75%) studies [40-42] used the Tesserae data set [63]. Compared with students and adults, the employee population was the least studied, with the fewest articles. However, the studies with employees had the largest number of participants, with a mean of 427.3 (SD 280.3). All 4 studies used regression-based predictive modeling, and 2 (50%) of them [40,56] evaluated a variety of models, with logistic regression, support vector machine, and random forest being the most common methods. Detecting and predicting employees’ stress in workplaces were examined in tandem with employees’ work performance. The research goal for these studies was to understand the underlining reasons for lowered work-related productivity. In contrast to the other 2 populations (ie, students and adults), less mobility was seen as positive for employees because less mobility in workplaces was associated with more positivity and higher performance.

**Table 5:** Summary of the reviewed studies with employee participants.

Study, year	Aim	Data collected	Operating system	Behavioral patterns	Predictive modeling	Verification surveys	Sample size, n
Mirjafari et al [56], 2019	Predict stress and job performance	Accelerometer, GPS, phone lock and unlock, and light sensor data	iOS and Android	Higher performers unlocked their phone fewer times during evenings, had less physical activity, visited fewer locations on weekday evenings, were more mobile, and visited more locations during weekends.	Evaluated logistic regression, support vector machine, random forest, and XGBoost^a^ in terms of employee performance classification; XGBoost was the best model with 5-fold cross validation.	ITP^b^, IRB^c^, OCB^d^, and CWB^e^	554 employees
Nepal et al [40], 2020	Detect stress, well-being, and mood	GPS, phone lock and unlock, accelerometer, Bluetooth, and phone use data	iOS and Android	Promoted employees spent more time on their phones during early mornings and late evenings and had more unlocks during the night time than nonpromoted employees. Women’s mobility increased after promotion, whereas men’s mobility decreased.	Evaluated logistic regression, support vector machine, Gaussian naive Bayes, random forest, and k-nearest neighbor in terms of their classification between promoted and nonpromoted periods; the best model was logistic regression trained on ROCKET^f^-based features.	CWB, OCB, IRB, and ITP	141 employees
Saha et al [41], 2019	Predict stress and workplace performance	Light sensor, GPS, accelerometer, and phone lock and unlock data	iOS and Android	Stress was higher with increased role ambiguity.	Linear regression was used to predict a well-being score.	IRB, ITP, and OCB	257 employees
Morshed et al [42], 2019	Predict mood instability	Light sensor, GPS, accelerometer, and phone lock and unlock data	iOS and Android	Mood instability was negatively correlated with the duration of sleep, the number of conversations, the amount of activity, and outdoor mobility.	Ridge regression with regularization was used to infer a mood instability score.	EMAs^g^, PAM^h^, and PANAS^i^	757 employees

^a^XGBoost: extreme gradient boosting.

^b^ITP: Psychological Type Indicator.

^c^IRB: in-role behavior.

^d^OCB: organizational citizenship behavior.

^e^CWB: counterproductive work behavior.

^f^ROCKET: random convolutional kernel transform.

^g^EMA: ecological momentary assessment.

^h^PAM: Patient Activation Measure.

^i^PANAS: Positive and Negative Affect Schedule.

### Passive Sensors

#### Overview

Table 6 provides an overview of the range of sensors used to detect patterns related to mild mental health symptoms and summarizes the evidence of the effectiveness of the various sensors. The first column lists the sensor, and the second column presents how the data from that sensor are interpreted; in other words, it presents the behavior-related information that the sensor data are intended to represent. The third column indicates which articles found significant associations between the specific sensor and stress, anxiety, or mild depression. The fourth column indicates which articles found no significant associations between the specific sensor and mental health outcomes (ie, explicitly stated so in the articles). In the subsequent sections, we discuss the types of activities detected by the sensors.

**Table 6:** Sensor summary of the reviewed studies.

Sensor	Behavior	Evidence for effectiveness	No evidence
GPS	Location and physical activity	[27,30,35,37,39-42,44-53,56-61]	[28,31,55]
Microphone	Voice recognition, ambient sound, and sleep	[26,27,32,39,42,43,45,48,50,52,60]	[28,41,47]
Light sensor	Time spent in darkness and sleep	[36,39,41,43,45,50,52,56,58-61]	[28,42,48]
Accelerometer	Movement and physical activity	[27,30,35-37,39-43,45-47,49-51,53,56-61]	[31,33]
Phone locks and unlocks	Phone use	[34,35,39,40,43,45-47,50,53,56,58-61]	[28,41,42]
Call logs	Social interaction and incoming and outgoing calls	[27,33,34,37,44-46,51,53,60]	[28,30,31,35,47,48,55]
Bluetooth	Social interaction	[40,42,43,46,47,60]	[28,51]
Wi-Fi	Indoor location	[27,38,42,47,60]	None
Keyboard	Typing patterns and muscle activity	[29]	[28]
SMS text messages and emails	Social interaction and incoming and outgoing messages	[27,32,33,37,44,45,52]	[28,30,31,48,55]
App use	Phone use and social media	[28,35,36,40,43,45,48,49,61]	None
Screen on and off	Phone use	[40,45,46,49,50,52,53,55,57]	[51]
Gyroscope	Orientation of the smartphone	[36,60]	None

#### Social Interaction: Call and Text Logs, Audio, Microphone, and Bluetooth

The social interaction of an individual is reflective of their current mood and mental state [44,64,65,66]. Individuals with depression and stress may be expected to decrease their social interactions. This is measured through the frequency of receiving texts and calls, how fast individuals respond, and the frequency of being around others. Among the 40 included studies, 18 (45%) [27,28,30,31,33-35,37,44-48,51,53,55,60] examined call logs to understand social interaction patterns, mainly through the number of incoming and outgoing calls, the number of missed calls, and the duration of calls. Individuals who experience depression and stress may engage in longer outgoing calls [51]. Evening communications were predictive of depression [47], anxiety, and loneliness [54]. Students who experienced discriminations [53] and anxious participants had more evening communications [54]. Metadata on SMS text messages were examined in 10 (25%) [27,28,30,31,33,37,44,45,48,55] of the 40 studies, including the frequency of receiving SMS text messages and the average time of responses. People who are socially anxious were found to take different amounts of time to respond to SMS text messages and calls [33]. Increases in the number of calls were associated with increased social anxiety [48]. Those who experienced social anxiety were less likely to call or text in public [44]. For students, fewer conversations were associated with more stress [39] and more mood instability [42]. One of the studies found that more emotionally unstable individuals tended to text more than emotionally stable individuals [27].

#### Location: GPS, Bluetooth, and Wi-Fi

Location data can provide insights into individuals’ mental health state in terms of the normal or abnormal variety and frequency of locations visited [67]. As presented in Table 6, GPS has been one of the most commonly used passive sensors for stress, anxiety, and mild depression research. The findings regarding location consistently demonstrate that students and adults who experienced depression, anxiety, or stress tended to visit fewer places [39,44,50,58-60]. One of the studies [48] found that location data are highly inversely correlated with mild depression severity. The main way in which this is measured is through the frequency of exiting the house, the variety of locations visited, and mobility. The frequency of exiting the house is less for individuals who are depressed, and there is less variety in the visited locations for individuals who are socially anxious. Individuals who are feeling depressed often experience being less energetic [68,69]. Overall, negative emotions were associated with time spent at specific locations, but this is also affected by personal routines and preferences [30]. For students, stress and lower subjective well-being were associated with more time spent on campus [39,49] and less time spent at campus food locations [39]. Students who experienced depression spent more time at home [60], whereas individuals at higher risk of psychosis spent less time at home [51]. Time spent at exercise locations was positively correlated with changes in depressive symptoms [48]. Another study [38] distinguished between students experiencing severe stress and those with normal stress levels, revealing that students with severe stress spent significantly less time on campus and were less involved in work-related activities compared with their counterparts with normal stress levels. As for employees, higher performers were found to visit fewer locations on weekday evenings but more locations during weekends [56].

#### Voice Recognition: Audio

The microphone is used to measure audio data of speech and ambient noises. One of the studies [26] examined how people with stress speak by analyzing their voice, including the speed of speech, how energetic their vocality is, and the pitch. One caveat is that the study by Adams et al [26] used audio captured within laboratory environments and found that stress could be recognized from the absence of speech. In variable environments, it will be harder to recognize the changing voice patterns. One study found that generalized anxiety and depression related to reward-related words in ambient speech, and social anxiety related to vision-related words [32]. Another study [52] identified that people with depression tend to speak less and use more death-related words.

#### Sleep: Accelerometer, Audio, and Illuminance

Sleep is highly correlated with individuals’ mental state [26,35,36,42,45-47,59,60]. Among the 40 included studies, 5 (13%) [35,46,52,60] found that more disturbed sleep correlated with more depressive symptoms. However, occasional sleep disturbance is not necessarily predictive. For example, for those with social anxiety, sleep disturbance might be positive because it suggests night-time activity and social interactions. Metadata on the time spent in darkness can be indicative of sleep patterns. The study by Fukazawa et al [36] stated that anxiety levels increase when the time spent in darkness increases. The study by Di Matteo et al [52] found that individuals with symptoms related to social anxiety and depression spent less time in darker environments. Another study [39] stated that stress changed students’ sleep patterns, where they became less likely to move around between 6 PM and midnight. Of the 40 studies, 6 (15%) found that shorter sleep duration was correlated with more mood instability [42], more depressive symptoms [59,60], and more stress [36,44]. One of the studies [45] also found that the student population, in general, tended to sleep less during examination periods and slept more during breaks, and they felt more stressed during both breaks and examination periods.

#### Phone Use: On and Off Screen, Lock and Unlock, and App Use

Today, smartphones are used for self-regulated “distractions,” such as the use of social media [38]. This type of self-regulated distraction can temporarily reduce stress. The study by Chikersal et al [47] showed that depression can impact concentration levels, so if distraction by phone can be measured, this could be a potential predictive marker. Several studies found that increasing phone use was correlated with more depressive symptoms [46,47,50,52,58-60], anxiety [52,59], impulsivity [34] and lower subjective-wellbeing [49]. The study by Morshed et al [42] outlined that for postsemester depression, phone use at night is not predictive, whereas another study [47] summarized that phone use during the day is predictive of depression. More frequent phone locks or unlocks correlated with higher levels of depressive symptoms [60] and impulsivity [34]. Higher performing employees tended to unlock their phones less frequently in the evenings [56]. Additionally, individuals who were promoted spent more time on their phones during early mornings and late evenings, with more unlocks occurring during nighttime compared with their nonpromoted counterparts [40].

#### Physical Activity and Mobility: Accelerometer

According to Table 6, along with GPS, accelerometer is one of the most widely used passive sensors in digital phenotyping research to monitor participant’s mobility, activity, and sedentary periods. Increased sedentary time was correlated with increased depressive symptoms [47,48,50,58-60], increased mood instability [27,42], increased stress [36] and decreased subjective well-being [49]. Exercise duration was positively correlated with changes in anxiety [36] and depressive symptoms [48]. The study by Mirjafari et al [56] found that the amount of movement and physical activity was related to employee’s stress level and highlighted that if the activity is regular, it should reduce stress. Different occupations require different levels of physical activity, social interactions, and mobility. For instance, developers spend most of their time at their desks, and their tasks might require less social interaction and mobility at work, but this does not mean they are more stressed. Project managers have more mobility during the day, and this may be because they need to move around to meet with the stakeholders [56]. Several studies have observed variations in mobility and gait consistency. The study by Boukhechba et al [44] reported that individuals with high social anxiety exhibited a narrower range of activities, whereas the study by Xu et al [60] revealed that students experiencing depression demonstrated more consistent mobility patterns. Additionally, accelerometer data indicated that individuals with low social anxiety maintained a steady walking pace, whereas those with high social anxiety tended to walk more rapidly and with greater irregularity [33].

#### Muscle Activity: Keyboard

Stress can cause muscle tension [70,71]. One of the studies [29] collected the data of users with stress via a keyboard in a laboratory environment and found that typing pressure significantly increased under stressful conditions.

### Challenges

Digital phenotyping for mild mental health symptoms in nonclinical participants can present ethical challenges, limitations to the research, and technical challenges. We review the challenges that were stated in the literature.

#### Ethical Challenges

Among the 40 included studies, 7 (18%) specifically mentioned privacy-related ethical concerns [28,31,35,36,40,41,43]. A major concern for participants across several studies was whether authorities, such as employers or teachers, will have access to their data. One of the studies [28] conducted in-depth interviews with 15 students to understand their perspectives on digital phenotyping through app prototypes. They found that the students’ core concerns were whether the acquainted university staff had access to the data. They also found that students’ acceptability of such apps depends on the perceived relevancy of the data collected and the effects on students’ devices. The study by Nepal et al [40] with employees reported a similar privacy concern of whether the employees’ data would be leaked to their boss; if the boss is aware of a potential mental health issue, it may impact their work performance ratings.

The methods of collecting and storing passive sensing data also present privacy concerns [28,70,72], particularly when the tracked data involve sensitive topics, such as mental health [72]. Sensors that infer individuals’ social interactions provide insights into their mental health status [26,36,53]. However, these types of data were less likely to be shared by participants because of privacy concerns. In the study by Rooksby et al [28], students identified camera, microphone, call log, and keyboard data as highly unacceptable types of data to capture.

Location data were associated with privacy and security concerns. In the study by Wen et al [34], participants felt uncomfortable with location tracking because it might breach their privacy and were hesitant to log their location when they moved from one place to another. Some studies excluded specific sensors to protect the participant’s privacy. Location data were not recorded owing to security concerns, even though they could provide valuable insights into the mental state [36,38]. In the study by Adams et al [26], the microphone was disabled to capture calls and conversations while individuals were talking to their family members. Another ethical concern was regarding the misuse of data. The main focus in studies of digital phenotyping using smartphones was on tracking participants’ usual behavioral patterns and identifying whether they behaved unusually. There were concerns regarding secondary uses. For example, participants’ leaked data can be used for advertising purposes or to create content [34,41].

#### Limitations to the Research

Coping mechanisms related to stress and anxiety vary among individuals [22]. Individual differences can make it challenging to label individuals as stressed, anxious, or depressed, particularly nonclinical participants. Certain behavioral patterns can be generally expected; however, not all individuals will follow the same pattern. To make generalizable and powerful analyses and understand behavioral patterns associated with mild mental health concerns, it is recommended to study diverse groups for longer than a 2-week period. Of the 40 included studies, 2 (5%) [33,39] focused on a particular demographic subset, namely, undergraduate students. Therefore, the generalizability of the studies is limited. In the studies by Rooksby et al [28], Exposito et al [29], and Wang et al [50], limited variation in representation was seen as a major limitation. The studies by Rhim et al [49], DaSilva et al [39], and Fukazawa et al [36] stressed the importance of selecting a wider age group, as younger people use their smartphones proactively, whereas older people’s behavioral patterns might show differences when they are experiencing mild mental health symptoms. The study by Nepal et al [40] suggested that diverse population testing is required for more reliable results, considering interindividual differences. Furthermore, the accuracy and effectiveness of machine learning models are highly affected by data set quality. We noticed that over the last 4 years [38,46,57,60], there has been increased focus on the generalizability of machine learning models, with the goal of assessing generalizability across students from various years, classes, and institutions.

#### Technical Challenges

Digital phenotyping studies on mild symptoms related to mental health with nonclinical participants presented technical challenges. A main concern was the accuracy of the sensor data collected from smartphones. The study by Fukazawa et al [36] sought to understand the time spent in darkness and its effects on the relationship between stress and anxiety patterns and sleep. However, when individuals carried their smartphone in their pockets or bags, the smartphone could not detect the darkness of the environment. This presented a challenge because illuminance data were captured even when the phone was not used actively. Similar concerns were raised in the study by Di Matteo et al [52]. The time spent in darkness feature did not distinguish whether the device was in a dark room or a dark location (ie, in the pocket). The study by Melcher et al [35] stated that the captured accelerometer data may not accurately represent daily activity, as not all participants constantly carried their phones throughout the day. In the study by Di Matteo et al [32], environmental audio did not produce clear transcripts in louder environments. This study mentioned that transcripts were produced based on dictionaries, so language analysis of complex speech, such as metaphors and sarcasm, was ignored. Therefore, the entire content of the conversation might not be correctly interpreted. In the study by Di Matteo et al [52], similar challenges were identified, as the speech data produced from smartphones were not clear. The recorded voices of the participants were masked by those of the people around them or even sound from other sources such as television or radio. Moreover, it was not possible to identify whether the death-related words came from the participants or from the people they interacted with.

Another technical challenge identified was battery life [47]. As expected, moment-by-moment data collection requires high power use, which might shorten the battery life. Participants had to charge their phones more often, which was inconvenient, and altered their usual behavior because they could not carry their phones as usual when the phones were charging. The study by Chikersal et al [47] mentioned another technical limitation: the transfer rate was affected if the app stopped working randomly. During these times, data were not transferred or collected. With the increase in the use of 5G technology, Wi-Fi data for indoor locations may cease to be relevant. In the study by Zakaria et al [38], some users were on their 5G indoors rather than their Wi-Fi, and this may point to a future trend of the use of 5G. We now turn to the discussion.

## Discussion

### Principal Findings

This literature review examined digital phenotyping studies that detected and predicted stress, anxiety, and depression in their mild states in nonclinical populations using data collected from smartphones. The primary objective of digital phenotyping in the context of mild mental health was similar among the 3 participant cohorts: students, adults, and employees. However, notable distinctions emerged among these groups. Among university students, the geographical proximity and relevance of the university campus were discerned as influential factors. Moreover, academic pursuits, particularly coursework and study-related activities, assumed significance within this demographic. Conversely, among employees, work aspects held salience, accompanied by the workplace environment. The remaining studies encompassed a general population cohort, delineated by undisclosed characteristics. Overall, we found that identifying behavioral abnormalities related to stress and anxiety was possible but raised certain challenges. Generalized stress and anxiety symptoms vary largely among individuals, whereas serious diagnoses, such as bipolar disorder or schizophrenia, have well-documented behavioral changes. Sleep was a strong predictor variable, yet some individuals tended to sleep more while they were stressed, whereas others lacked sleep under stress. This may be one of the reasons why there are fewer studies and reviews completed on stress and anxiety compared with studies on serious conditions such as bipolar disorder, severe depression, and schizophrenia. Another reason is that clinical psychologists and psychiatrists who are familiar with clinical populations are leading the digital phenotyping research.

Studies tended to use self-report to categorize nonclinical populations as stressed, depressed, or anxious. It was not always clear whether the identified patterns of the passive sensor data would effectively discriminate among groups. Most studies used prestudy and poststudy surveys to identify participants’ mental state. There were concerns raised regarding the accuracy of the categorization of self-report surveys. For instance, the study by Sefidgar et al [53] stated that students with stress may not report themselves as very stressed. Melcher et al [70] conducted a review and found that students were concerned regarding their professors learning about their data [71]. Thus, the accuracy of self-report remains an issue for passive sensing studies that use self-report labels, especially when there are privacy concerns. This may be related to the high dropout rates in the studies.

Many types of data sensors were used in the reviewed studies. Few articles related sensor patterns to specific symptoms validated by relevant psychological evidence. One of the studies [46] extracted interpretable rules (such as intermittent sleep episodes or number of bouts of being asleep or number of outgoing calls during weekends) through association rule mining to distinguish the behavioral patterns between students who were depressed and students who were not. However, although the behavioral patterns were identified, they were not validated to be exclusive to the addressed mental health issue; for example, high mobility and physical activity do not necessarily mean that the person is not stressed. In the study by Tseng et al [45], students were more mobile during the examination week, despite being under high pressure and stress. In the same study, some students were less mobile when studying for their examinations, which we cannot necessarily be interpreted as being under stress. Of the 40 included studies, 4 (10%) [35,58,59,61] explored the effects of the COVID-19 pandemic on behavioral and mental health. Additional recent investigations, which independently gathered their own data sets during the COVID-19 pandemic, have shown that quarantine measures have influenced individual behavioral patterns. For the purpose of making precise predictions in digital phenotyping, it is imperative to consider contextual and environmental factors.

Privacy and secondary data uses were the main concerns identified for digital phenotyping. Individuals using digital phenotyping systems have the right to provide informed consent. This means that they should be made aware of how all their data will be used, who will have access to their data, where their data will be stored, and for how long their data will be stored, and they have the right to decline to participate. We urge researchers and medical practitioners to carefully consider the system design and requirements because data transferred to the cloud and other services may fall under various service agreements. To empower end users and improve the quality of digital phenotyping systems, we recommend that transparent algorithms and explainable artificial intelligence be combined with user-accessible and understandable displays so that adults can engage in the process of identifying and categorizing patterns related to mild mental health symptoms.

The digital phenotyping research focused on in this review may enable the design of tailored intervention programs for nonclinical participants who are showing symptoms of stress, anxiety, and mild depression. Most of the studies included in this review were conducted within a restricted timeline and limited scope of detection and prediction. Only 4 (10%) of the 40 studies mentioned potential intervention programs upon predicting stress, anxiety, and mild depression [31,38,47,53].

Our review has some limitations. We excluded studies conducted with teenagers, children, and adults who were clinically diagnosed. Thus, we missed studies that focused on the detection and prediction of stress, anxiety, and mild depression in these populations. These populations are likely to show different patterns than those in adults who are not clinically diagnosed. Further, we excluded studies conducted using technologies other than smartphones. We chose this more limited subset of technologies to scope findings related to widely available technologies. The availability of technologies is changing rapidly, and wearables such as smartwatches are becoming more common. As wearable technologies become ubiquitous, we recommend including them in future systematic reviews.

This literature review is unique in that it examines studies focused on the behavioral patterns of nonclinical populations, namely students, employees, and adults who are stressed, anxious, or mildly depressed. We examined each type of sensor and indicated when it was significantly associated with mild mental health symptoms. We identified commonalities in the studies in terms of ethical challenges, limitations to the research, and technical challenges.

### Conclusions

This systematic literature review found that digital phenotyping can be an effective way of identifying certain behavioral patterns related to stress, anxiety, and mild depression. A range of passive sensors was used in the studies, such as GPS, Bluetooth, ambient audio, light sensors, accelerometers, microphones, illuminance, and Wi-Fi. We found that location, physical activity, and social interaction data were highly related to participants’ mental health and well-being. The surveyed literature discussed the ethical and technical challenges that limit the accuracy and generalizability of results. One of the greatest challenges was privacy concerns, and these were primarily related to camera, location, SMS text message, and call log data. Another challenge was the significant variation among individuals and their unique behaviors related to mental health. Finally, technical limitations have not been fully resolved, with issues such as the sensor for illuminance still capturing data while not in use reducing the accuracy of the collected data. It is hoped that this overview of digital phenotyping and mental health studies conducted in the last decade, including the common privacy and technical concerns, can help move this area of research forward, ultimately improving the quality of passive sensing, and provide benefits in terms of the early detection of relevant mild mental health phenomena.

## Appendix

Multimedia Appendix 1 The PRISMA 2020 checklist.

## Abbreviations

PRISMA: Preferred Reporting Items for Systematic Reviews and Meta-Analyses
